# Direct esterification of sucrose: novel lead-compounds against cancer

**DOI:** 10.1080/07853890.2021.1896911

**Published:** 2021-09-28

**Authors:** Luís Filipe, Krasimira Petrova

**Affiliations:** Departamento de Química da Faculdade de Ciências e Tecnologia, Universidade Nova de Lisboa, Caparica, Portugal

## Abstract

**Introduction:**

Phenylpropanoic sugar esters are a class of naturally occurring active substances, mainly found in ancient oriental medicinal plants (Figure 1), with a scope of activity ranging from antioxidant, antibacterial as well as anti-tumoral [1]. Though relatively simple in structure – owing to their resemblance to natural carbohydrates - few have been the successful attempts to obtain them in the laboratory, hampered mainly by the fact that the high similarity between functional groups in sugars restrains the feasibility of current methods and their reproducibility in industrial scales [2]. Our study aims for the development of reliable and effective methods for the synthesis of a library of phenylpropanoic esters derived from sucrose.

**Materials and methods:**

The lead-compounds are obtained by treating sucrose with a vigorous basic medium. The preferential esterification in the sugar hydroxyls is catalysed by a metal halide salt. The different compounds obtained are meant to be tested and screened for their biological applications, with particular emphasis on their action against cancer cells.

**Results:**

Our most appealing discovery is the possibility of directly enhancing the regioselectivity of the 2, 3′, 6 and 6′-hydroxyl positions in the sugar moiety induced by transition-metal salts as catalysts [3] (Scheme 1). Different metal salts induce the different hydroxyls mentioned for a direct esterification with the acylating agent, which reflects the specificity and versatility of this novel class of reactions.

**Discussion and conclusions:**

We can conclude, so far, that cobalt and copper chloride salts give the best results in terms of yield (more than 30% conversion) and decreased reaction time (less than one hour). To further extend our knowledge about the metal-chelate directed acylation method, we are also experimenting with other halide salts, namely zinc, calcium, nickel and iron, as well as with derivatized molecules of sucrose. Thus, our work can certainly lead the development of novel drugs and resourceful cancer therapies.

**Figure 1.**
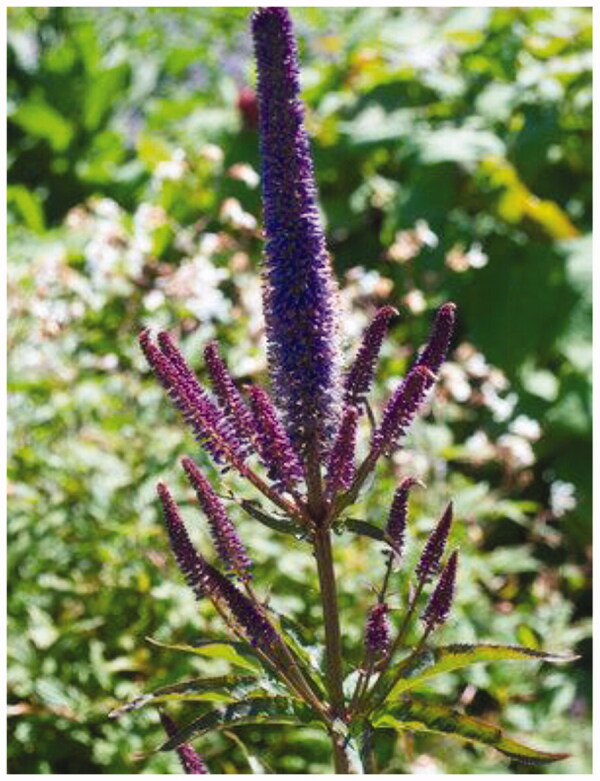
*Veronicastrum Sibiricum*, one of the plants containing phenylpropanoic sucrose esters as the active substance.

**Scheme 1.**
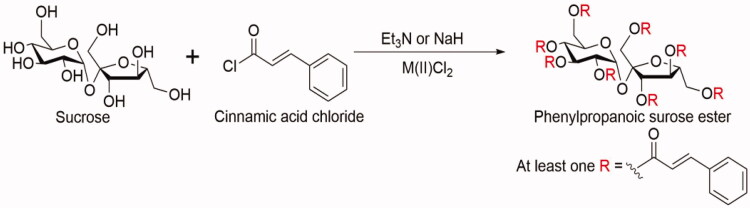
General route for the metal-chelate direct esterification of sucrose molecule.
